# Supplementary material for: Identification of photoperiod-induced specific miRNAs in the adrenal glands of Sunite sheep (Ovis aries)
Source: Front Vet Sci. 2022 Jul 22;9:888207. doi: 10.3389/fvets.2022.888207 (PMC9354845; doi:10.3389/fvets.2022.888207)
Supplement: Supplementary file 1 [file Data_Sheet_1.ZIP › Supplementary file/miRDeep2 file.zip!/miRDeep2 file/Know_Structure/SPLP42A3/oar-mir-17.pdf]

miRBase precursor : oar-mir-17
Total read count : 1767
oar-miR-17-5p read count : 1724
remaining reads : 43

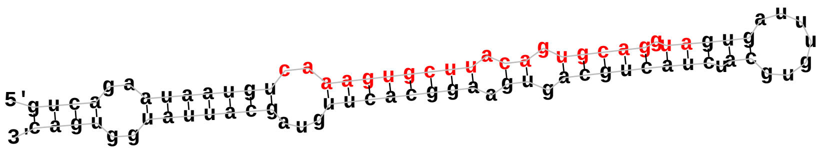

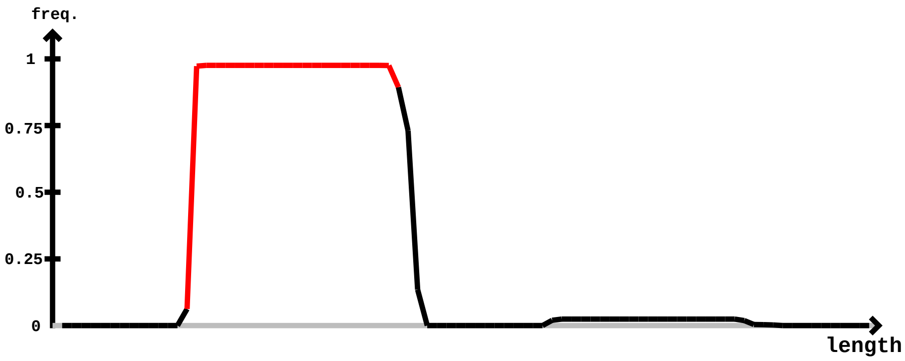


```
                oar-miR-17-5p
5'- gucagaauaaugucaaagugcuuacagugcagguagugauuugugcaucuacugcagugaaggcacuuguagcauuauggugac   -3'    exp
    ((((..(((((((..((((((((.((.(((((.(((((.......)).)))))))).)).))))))))...)))))))..))))    reads    mm    sample
    ............ucaaagugcuuacagugcaggu..................................................    24       0     seq
    ............ucaaagugcuuacagugcaggua.................................................    48       0     seq
    ............ucaaagugcuuacagugcagguag................................................    33       0     seq
    ............ucaaagugcuuacagugcagguagu...............................................    5        0     seq
    .............caaagugcuuacagugcagg...................................................    1        0     seq
    .............caaagugcuuacagugcaggu..................................................    119      0     seq
    .............caaagugcuCacagugcaggu..................................................    1        1     seq
    .............caaagugcuCacagugcaggua.................................................    3        1     seq
    .............caaagugcuuacagugcaggua.................................................    235      0     seq
    .............caaagugcuCacagugcagguag................................................    6        1     seq
    .............caaagugcuuacagugcagguag................................................    1011     0     seq
    .............caaagugcuCacagugcagguagu...............................................    2        1     seq
    .............caaagugcuuacagugcagguagu...............................................    231      0     seq
    ..............aaagugcuuacagugcaggua.................................................    1        0     seq
    ..............aaagugcuuacagugcagguag................................................    3        0     seq
    ..............aaagugcuuaUagugcagguag................................................    1        1     seq
    ..................................................acugcagugaaggcacuugu..............    10       0     seq
    ..................................................acugcagugaaggcacuugua.............    25       0     seq
    ...................................................cugcagugaaggcacuugua.............    1        0     seq
    ...................................................cugcagugaaggcacuuguag............    1        0     seq
    ...................................................cugcagugaaggcacuuguagc...........    2        0     seq
    ...................................................cugcagugaaggcacuuguagca..........    4        0     seq
```
